# Palatal Rugae Patterns in Fars, Turkmen, and Sistani Ethnicities in the Eastern Part of the Caspian Littoral of Iran

**DOI:** 10.3390/diagnostics13020200

**Published:** 2023-01-05

**Authors:** Donya Rahebi, Aliakbar Naghavialhosseini, Mina Pakkhesal, Abdolhalim Rajabi, Fatemeh Mirzaei, Nesreen A. Salim, Malik Sallam

**Affiliations:** 1 Dental Research Center, Golestan University of Medical Sciences, Gorgan 49138-15739, Iran; 2 Orthodontics Department, School of Dentistry, Golestan University of Medical Sciences, Gorgan 49138-15739, Iran; 3 Community Oral Health Department, School of Dentistry, Golestan University of Medical Sciences, Gorgan 49138-15739, Iran; 4 Department of Health Management and Social Development Research Center, Faculty of Health, Golestan University of Medical Sciences, Gorgan 49138-15739, Iran; 5 Student Research Committee, Golestan University of Medical Sciences, Gorgan 49138-15739, Iran; 6 Prosthodontic Department, School of Dentistry, The University of Jordan, Amman 11942, Jordan; 7 Prosthodontic Department, Jordan University Hospital, Amman 11942, Jordan; 8 Department of Pathology, Microbiology and Forensic Medicine, School of Medicine, The University of Jordan, Amman 11942, Jordan; 9 Department of Clinical Laboratories and Forensic Medicine, Jordan University Hospital, Amman 11942, Jordan

**Keywords:** forensic odontology, hard palate, forensic marker, morphology, palatine rugae analysis

## Abstract

In forensic medicine, it is important to identify whole or fragmented bodies. This aim can be particularly challenging in mass disasters. Palatal rugae patterns can be used as a surrogate parameter in forensic medicine. This stems from the difficulty in falsifying these patterns, their resistance to trauma, to decomposition for several days postmortem, and to combustion under high-temperatures, as well as being distinguishable among different races. The present study aimed to analyze the differences in the palatal rugae patterns among three Iranian ethnicities (Fars, Turkmen, and Sistani). This retrospective study involved the use of archived materials. The study casts were selected from the database of patients who visited a private orthodontics clinic. A total of 309 dental casts (103 Fars, 103 Turkmen, and 103 Sistani) were assessed, which belonged to 181 females and 128 males aged between 12 and 30 years (mean: 16.86 ± 3.18 years). The difference in the mean number of palatal rugae in women between the three ethnicities was statistically significant. Also, the differences in the total number of straight rugae were significant between the three ethnic groups. The most common rugae shapes in the three ethnic groups were the straight and wavy shapes. The length of the palatal rugae in the primary and secondary rugae among the study subjects younger than 18-years-old was significantly different between the three ethnic groups. Thus, the present research highlighted the differences in palatal rugae patterns among three Iranian ethnicities. Therefore, palatal rugae can be used in forensic medicine as a complementary approach to human identification.

## 1. Introduction

Human forensic identification depends on distinctive characteristics of individuals and systematic procedures to identify these characteristics [1,2,3,4]. A branch of dentistry and forensic medicine is forensic odontology, which involves the utility of dental evidence in the form of antemortem and post-mortem dental records to serve human identification purposes [5,6,7]. Unique individual identities can be related to dental characteristics such as: (1) tooth morphology; (2) variability in teeth size/shape; (3) restorations; (4) pathologies; (5) missing tooth; (6) wear patterns; (7) teeth crowding; (8) variation in teeth color and position; (9) rotations among other distinct teeth anomalies; and (10) unique palatal rugae (plica palatine) patterns [5,8,9,10,11]. Additionally, an examination of dental features can be a valuable tool supplementing fingerprinting and DNA profiling to determine age, sex, and race/ethnicity, among other variables, even in the absence of antemortem dental records [5,12]. Furthermore, prosthetic dentistry can play an important role in supplementing forensic medicine to achieve more accurate, reliable, and investigatory data [13,14]. The prosthetic dentistry arsenal to aid in forensic medicine, as presented by Chugh and Narwal [15], includes palatoscopy (palatal rugoscopy) to identify palatal rugae patterns, cheiloscopy to identify lip prints, bite marks, fixed prostheses, and implants, as well as denture marking [16,17,18].

Human identification can be particularly challenging during mass disasters (e.g., earthquakes, floods, terrorist attacks, etc.) [19,20,21,22]. However, disaster victim identification (DVI) is an essential step for certification of death as well as for personal, social, and legal purposes [23]. The most commonly used approaches for DVI include DNA profiling, fingerprints, and dental record comparisons [24,25,26,27]. However, constraints challenge the use of some methods (e.g., fingerprints) in situations where the hands are mutilated or charred [5,28,29].

The palatal rugae comprise the part of the mouth roof located behind the incisive papillae and in front of the hard palate and develop from connective tissue that covers the maxillary bone in the twelfth to fourteenth weeks of intrauterine life [6,30,31]. The palatal rugae length changes during palatine growth, but their position/ shape remains unchanged for life [32,33,34,35].

The length of palatal rugae can be classified into three categories: (1) rugae more than 5 mm in length which are considered as primary rugae; (2) rugae of 3–5 mm in length, which are classified as secondary rugae; and (3) rugae of 2–3 mm in length which are considered as fragmentary rugae, with the rugae less than 2 mm in length being disregarded [36]. Moreover, the shape of individual rugae, as classified by Thomas and Kotze, is described as straight, wavy, curved, circular, and angled [36].

The palatal rugae patterns’ stability changes during orthodontic treatment, tooth extraction, and finger sucking during childhood [32,37]. Palatal rugae patterns are resistant to diseases, chemical aggression, fires, and trauma, as well as its resistance to decomposition after death. This is related to the protection of palatal rugae by the surrounding tissues including the lips, tongue, bone, and teeth, besides the buccal fat pads [37,38,39]. The view of palatal rugae as an equivalent to fingerprints is related to its uniqueness in an individual, as well as its uniqueness in shape and structure [30,31]. Despite the similarities in the palatal rugae patterns among twins, they are highly individualistic [30,32,37].

Therefore, the palatal rugae can be useful in forensic investigation due to their uniqueness, stability, and post-mortem resistance to decomposition [30,32,37,40]. The most common forensic identification methods are the use of fingerprints, DNA profiling, and dental records, but these techniques suffer from various limitations [6,35,38]. Thus, palatoscopy can be useful as a complementary identification tool in cases where it is impossible or difficult to apply other medico-legal identification tools, including fingerprints, DNA profiles, and dental records [38,39,41,42].

Palatal rugae vary in terms of shape, length, and direction among populations and between individuals [30,31]. So, palatal rugae variability can be viewed as a specific feature among different ethnic groups [32]. An ethnicity is a group of people who are identified together based on similarities including the ancestral, linguistic, socio-cultural, or national characteristics [38]. The utility of palatal rugae as a forensic tool is of particular importance in mass forensic investigation, such as DVI, in which people from different ethnicities are subjected to forensic identification [16,43,44]. Previous studies showed the value of such an approach, with significant differences in palatal rugae patterns among different ethnicities [6,30,45,46].

In Iran, Golestan Province is located in northeastern part of the country, southeast of the Caspian Sea. The total area of Golestan Province is 20,893 km^2^, which forms about 1.3% of Iran’s total area. The neighborhood of Golestan Province, with some regions of different cultural backgrounds, has contributed to the heterogeneous ethnic mixture observed in the province [47]. Agriculture is the main occupation in the rural area of Golestan, which is inhabited by different ethnic groups. The major ethnicities within Golestan Province are estimated as follows: Fars (40%), Turkmen (32%), Sistani (also called Zaboli) (15%), and Azeri Turks (5%) [48]. The Turkmen is the ethnic group that migrated from central Asia more than three centuries ago and live a traditional life, with prevalent intra-familial marriages. The Sistani group emigrated from southeastern Iran half a century ago [49,50]. Few studies have evaluated palatal rugae patterns among Iranian ethnicities, and none in the Golestan population [6,38]. Overall, palatal rugae patterns have been utilized in various disciplines, including: (1) comparative anatomy; (2) genetics; (3) forensic odontology; (4) prosthodontics; and (5) orthodontics [51]. One of the most important applications of palatal rugae patterns is personal identification in the field of forensic odontology [44,52]. In addition, palatal rugae can be used as a landmark in the diagnosis and treatment planning during orthodontic treatment and in prosthodontics, besides its use to aid in speech and mastication by using palatal prostheses that incorporate the palatal rugae [42,53,54,55]. In light of the need for more evidence to justify the utilization of palatal rugae patterns in dentistry, the present study was conducted to examine the possible differences in palatal rugae patterns among different ethnicity residents in Golestan Province, Iran. Therefore, in this study, we evaluated the patterns of palatal rugae among the Turkmen, Fars, and Sistani ethnicities of Golestan Province to investigate whether palatal rugae patterns can be used to identify the ethnicity and uniqueness of individuals for potential application in forensic medicine.

## 2. Materials and Methods

### 2.1. Study Design

The present study was based on a retrospective design involving the use of archived materials. The study casts were selected from the database of patients aged between 12 and 30 years, who visited a private orthodontics clinic in Golestan Province, Iran. The sample size was calculated as 309 pre-treatment casts, using the formula for multiple groups, based on the parameters presented in the Sheikhi et al. study [38], with a 0.050 level of significance (α), and 0.80 power of the study.

A total of 309 pre-treatment orthodontic maxillary cast models were analyzed, consisting of 103 casts in each group of the three different ethnic populations of Golestan Province (Fars, Turkmen, and Sistani). All the selected casts were made of high-strength dental plaster using maxillary alginate impressions, free of air bubbles or voids, especially in the anterior third of the palate. The casts belonged to individuals who were born in Golestan with recorded demographic characteristics (age, sex, and ethnicity) in their files and without a history of orthodontics treatment.

The present study was approved by the Research Ethics Committee of Golestan University of Medical Sciences (reference number: IR.GOUMS.REC.1398.377).

### 2.2. Data Gathering

The rugae shape and length measurements were performed by an individual researcher (D.R.) who was trained under the supervision of a qualified orthodontist. In the initial study, 20 casts were reassessed by the main examiner (D.R.) four weeks following the first assessment to determine intra-observer reliability. Those 20 casts were not included in the main study. Also, Cohen’s kappa test was used to evaluate the intra-observer reliability. The kappa score (0.89) indicated excellent agreement. 

The ruga classification (length and shape) was recorded based on the categories given by Thomas and Kotze [36]. The rugae outlines on the casts were delineated using a sharp black graphite pencil under adequate light and magnification using a handheld magnifying lens. Then, the number of rugae on either side of the midline was counted.

**Figure 1:**
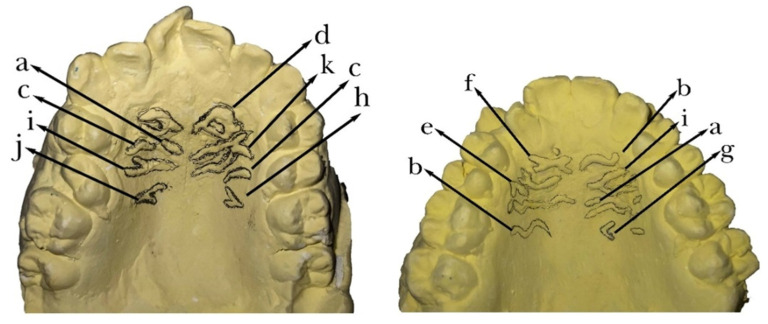
Different types of palatal rugae shape delineated in maxillary casts (a) straight; (b) wavy; (c) curved; (d) circular; (e) angle; (f) cross; (g) diverging; (h) converging; (i) branching with divergence; (j) branching with convergence; (k) non-specific.

The patterns of the rugae on each side to their morphology were determined and classified according to the following patterns: (1) straight, (2) curved, (3) angle, (4) wavy, (5) circular, (6) diverging (two rugae that originate from a common point medially and diverge away from the mid-palatal line), (7) converging (two rugae with different origins medially, joining on a common point laterally), (8) branching with divergence (one ruga with two or more branches directed away from the mid-palatal line), (9) branching with convergence (one ruga with two or more branches directed toward the mid-palatal line) and (10) non-specific (Figure 1). The rugae lengths were measured on each side using a Vernier caliper, calibrated to 0.01 mm. 

Based on their lengths, the rugae were divided into primary, secondary, fragmentary, and rugae with lengths of less than 2 mm, which were disregarded. Also, the lengths of the palatal rugae between the target ethnicities were analyzed in two groups: younger-than-18-years and older-than or equal-to-18-years, due to the elimination of the effect of growth on the lengths of rugae in terms of age. Each primary ruga’s direction was classified according to the angle between the line joining its origin and termination with a line perpendicular to the median raphe. The forward-directed rugae were associated with positive angles, the backward-directed rugae were associated with negative angles, and the straight-directed rugae were associated with parallel angles.

### 2.3. Statistical Analysis

All the statistical analysis was performed using STATA version 16 (Stata Corp LP, College Station, TX, USA). The descriptive statistical analysis was performed using STATA to obtain the means, standard deviation (SD), and frequency from each set of category data. The Mann–Whitney U test was used to assess the significant difference in the total number of each type of palatal rugae between males and females. The chi-squared test was used to determine the distributions of morphology and direction of the palatal rugae in the ethnic groups. A one-way ANOVA was used to compare the lengths of the palatal rugae among the ethnic groups, followed by Tukey’s post hoc test. The level of statistical significance was set at *p* < 0.050.

## 3. Results

In this study, 309 dental casts were examined (103 Fars, 103 Turkmen, and 103 Sistani) belonging to 181 (58.57%) females and 128 (41.43%) males, aged between 12 and 31 years (mean ± SD: 16.86 ± 3.18) (Table 1).

As shown in (Table 2), only the difference in the mean number of palatal rugae in females among the three ethnicities population was statistically significant (*p* = 0.010).

According to the post hoc test, the mean number of palatal rugae in the Sistani women was significantly lower than in the Turkmen women (*p* = 0.017) and the Fars women (*p* = 0.017), but there was no significant difference between the Fars and Turkmen women (*p* = 0.990). However, the mean number of palatal rugae on the left side of the women’s palates between the Fars (6.5 ± 1.40), Sistani (5.7 ± 1.54), and Turkmen (6.15 ± 1.24) ethnicities did show significant differences (*p* = 0.008). Additionally, and based on the post hoc test, the mean number of palatal rugae on the left side for women of Turkmen ethnicity compared to Sistani (*p* = 0.315) and Fars (*p* = 0.414) was not significant, but there was a significant difference between the Fars and Sistani females (*p* = 0.008). However, the left side of the palate of the males showed significant differences between the ethnicity of the Fars (6.06 ± 1.31), Sistani (5.36 ± 1.48) and Turkmen (6.15 ± 1.64), (*p* = 0.008). According to the post hoc test, the mean number of palatal rugae on the left side for the Fars males was not significant compared to the Sistani (*p* = 0.139) and Turkmen (*p* = 0.990), but there was a significant difference between the Turkmen and Sistani males (*p* = 0.034) (Table 2).

In addition, as presented in (Table 3), the differences in the total number of straight rugae were significant between the three ethnicity groups.

**Figure 2:**
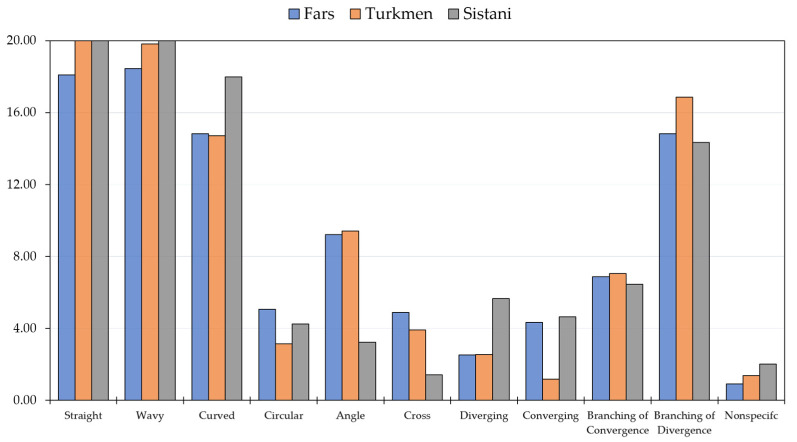
Frequency distribution of the different palatal rugae shapes in three ethnicities.

Consequently, according to the post hoc test, the mean number of straight rugae in the Turkmen was significantly higher than in the Fars and Sistani, but there was no significant difference between the Fars and Sistani. In addition, the mean number of wavy and curved-shape rugae was significantly different between the three ethnic groups. As shown in (Figure 2), the most common rugae shapes in the three ethnic groups were straight and wavy.

Moreover, as shown in (Table 4), there was no significant difference between the mean length of palatal rugae by ethnicity for the primary, secondary, and fragmented rugae in patients over 18 years old. However, in the study subjects younger than 18 years old, the lengths of the palatal rugae in the primary and secondary rugae were significantly different between the three ethnicity groups (Table 4).

In addition, the mean number in all three groups of primary, secondary and fragmented palatal rugae between the ethnicities in people older than 18 years, in both sexes and on both sides of the palate, was not significantly different. The distribution of the total number of rugae direction by sex and side is shown in (Table 5).

## 4. Discussion

Human identification, particularly in disasters or mass settings, is one of the most challenging tasks from a forensic point of view [27]. Various scientific methods have been used for human identification, including those pertinent to forensic odontology [12,56,57]. The palatal rugae exhibit properties such as individuality, stability, and postmortem resilience, making them a suitable instrument for forensic personal identification [1,40,52]. A recent meta-analysis found that combining dimensional and morphological evaluations of the palatal rugae could potentially improve personal identification accuracy [42].

The present study showed that the total number of palatal rugae in the Sistani ethnicity is significantly less than their number among the Turkmen and Fars ethnicities, but the results showed no significant differences between the ethnicities of the Fars and Turkmen. According to the Sheikhi et al. study, the population of Tehran has significantly fewer palatal rugae than the populations of Hamedan and Kermanshah [38]. The total number of palatal rugae in the Indian population was likewise much larger than in the Tibetan population [33]. Furthermore, Arora et al. discovered that the total number of palatal rugae in the Manipur population is higher than in Karnataka [58]. Kashima et al. discovered that the total number of rugae in Japanese children was larger than the number in Indian children [59]. It was also discovered that Indigenous Australians have a larger number of palatal rugae than the Caucasian population [60]. Thus, the earlier research demonstrated the usability of rugae numbers as a tool for distinguishing ethnic differences, which was validated by the current study’s findings.

Furthermore, in the current study, the Fars women had a higher overall number of palatal rugae than the men. Similarly, Selvamani et al. discovered that women have more palatal rugae on both sides of the palate than men [61]. In addition, Kalyani et al. discovered that the average number of rugae in females was higher than in males [62]. However, Ibeachu et al.’s study found no significant difference between the sexes [63]. The rugae pattern variety and their possible use for sex discrimination in different populations produced disparate results, due to individual differences and the complex influence of genetic, growth, and environmental factors on their morphology [64].

The current investigation found that the total number of palatal rugae in the Fars ethnicity was higher on the left side of the palate than on the right [63,65,66,67]. Furthermore, the total number of palatal rugae on the left side of the palate was smaller in the Sistani ethnicity compared to the Turkmen and Fars ethnicities, although there was no significant difference between these ethnicities on the right side. According to a study conducted by Saini et al. [68], the overall number of right-side rugae in the Northeastern Indian population was larger than in the Northern Indian population, but there was no significant difference on the left side. Silva-Sousa et al. discovered genetic variations linked to left–right asymmetry in the number of palatal rugae [69].

The present study exhibited that the number of straight rugae in the Turkmen ethnicity was higher than in the Fars and Sistani ethnicities. The number of wavy rugae in the Sistani ethnicity was lower than those among the Fars and Turkmen ethnicities. Additionally, the number of curved rugae in the Sistani ethnicity was higher compared to those among the Fars and Turkmen ethnicities. Other investigations confirm the differences in the number of palatal rugae forms between populations. Sheikhi et al. found that the number of straight rugae in Iran was lower than in Kurdistan and Kermanshah [38]. According to the same study, the population of Hamedan has more wavy rugae than the populations of Tehran and Kermanshah [38]. It was also discovered that the number of straight rugae in the Arab population was smaller than in India, while the number of wavy rugae in the Arab population was larger [70]. Another study discovered that the number of straight rugae in the Ikwerre population in Nigeria was higher than the Igbo population, and the number of wavy rugae in the Igbo population was lower [63]. According to a study conducted by Nayak et al., the number of straight rugae in the southern population of India was larger than in the west, but the number of curved rugae in the southern population was lower [71].

The present study showed that the average number of straight, wavy, and angular rugae in the males in three ethnic groups was significantly different. The straight and angular-shaped rugae in the Fars males were fewer than in the Turkmen males, and the wavy-shapes in the Sistani males less than in the Turkmen and Fars males. In addition, the number of curved rugae in both sexes showed a significant difference between ethnicities, so that in the Sistani men, it was more than those among the Turkmen and Fars men, and the same figure in the Turkmen women was less than in the Fars and Sistani women. According to another study [63], the number of wavy-shaped rugae in the Igbo was much higher than the Ikwerre in both genders. However, the number of curved-shape rugae in the Ikwerre was much larger in both genders than in the Igbo. The number of circular shapes was larger in the Igbo men than in the Ikwerre men, while the number of straight forms was higher in the Ikwerre women than in the Igbo women. Malaysian men had more curved, angular, and complicated rugae than Egyptian men, according to another study. Egyptian women showed fewer wavy rugae and more straight rugae than Malaysian women [46]. In India’s two populations, however, there was no variation in the number of rugae forms between men and women [71].

In the current study, the most common palatal rugae shapes were wavy, straight, curved, and divergent in the Fars, straight, wavy, and divergent in the Turkmen, and straight, wavy and curved in the Sistani, respectively. The wavy pattern was the most prevalent among the Igbo population [63]. In contrast, the rugae of the Ikwerre population are typically curved and straight. Sherif et al. also demonstrated that the maximum type of rugae in Egyptians was straight, curved, and wavy, but it was curved, wavy, and straight in Malaysians. In both populations, the circular form had the lowest number [46]. According to Chandra et al., the most common type of rugae in the Ranchi population was wavy, curved, and straight. Curved, wavy, and straight shapes were common in the Patna population [72]. The most prevalent shape in Arabs is wavy, curved, straight, and branching, while it was wavy, straight, curved, and branched in Indians, respectively [70]. The rugae form differences between ethnicities may be attributable to hereditary or environmental causes [64]. According to previous research, environmental factors have the least influence on rugae formation, with genetic background being the key predictor [73].

In the three primary, secondary, and fragmented groups, there was no difference in the length of palatal rugae between the Fars, Sistani, and Turkmen ethnicities. However, it was discovered that the quantity of secondary rugae was different between the Igbo and Yoruba; additionally, there was no difference between them in terms of primary and fragmented rugae groups [43].

The present study showed no difference between the number of palatal rugae, based on the three groups of primary, secondary, and fragmented, on the left and right and between men and women. However, Ibeachu et al. discovered that men have more primary rugae than women in the two populations tested [63]. Kalia et al. discovered that Mysorean males have more primary rugae than women [74]. Dohke et al. found that the quantity of secondary rugae results in substantial changes in rugae morphologies between sexes [75]. In the study of Selvamani et al., there was no significant difference between the genders of primary and secondary rugae [61] As a result, discrete factors such as rugae shapes can produce superior findings when comparing ethnicities [63]. Furthermore, the current study found that the maximum length of rugae is of the original type, which is consistent with the findings of most studies [38,60,65,67,70].

The current study revealed more of the forward-directed rugae in the Turkmen ethnicity than in the Sistani ethnicity. While Saini et al. found that the number of backward rugae in the Northeastern India population was greater than the population of North India [68].

The present study showed that the number of forward rugae in women of the Sistani ethnicity was less than in the Turkmen ethnicity. Also, the number of straight-directed rugae for women in the Turkmen ethnicity was less than in the Fars ethnicity. Similarly, it was shown that the rugae direction of Egyptian and Malaysian men differed significantly. This difference, however, was not detected in women between the two populations [46]. According to the findings of this study, the most prevalent rugae direction in all three ethnicities was backward-directed, followed by forward-directed, and straight-directed rugae. According to Mattoo et al., forward-directed rugae are the most common, followed by backward-directed rugae, and then straight-directed rugae [76].

The use of palatal rugae as an individual identification tool can be limited by the lack of availability of antemortem data [5,57]. Therefore, palatal rugae pattern data need to be supplemented by other individual identification characteristics, such as fingerprints, that have easily accessible and highly valid antemortem data [42]. Better availability of antemortem palatal rugae pattern data might be achieved by the use of optical 3D oral/dental scans that are utilized in modern dental practice [31]. Nevertheless, this approach should be complemented by methods for the proper recognition of patterns to take into account potential geometric changes in patterns that happen post-mortem [31]. As a result, digital data must be created, kept, and updated in order to describe the dental/oral geometry, and obtaining the data could be a standard dental service. The current constraints include a lack of adequate 3D scanners, corresponding design/fabrication tools, and staff qualified to operate them. Furthermore, for forensic purposes, the scanned data must be accessible afterwards, and hence must be saved in a suitable media, format, and access site [1,31].

The main limitation of the present study was the relatively small sample size. In addition, direct comparison with previously conducted research was difficult and limited, as a result of variations in the methodological approach and classification of palatal rugae. Moreover, dental casts were used to examine two-dimensional morphological patterns in this study; nevertheless, it is useful to assess palatal rugae using other techniques, such as stereoscopy and stereophotogrammetry, to explore the rugae in both three dimensions and the position of each ruga. Despite these limitations, this study is among the first in the literature to report on the palatal rugae pattern in three different ethnic populations in Iran, considering both the dimensions and the morphology of palatal rugae, and providing valuable baseline data and a preliminary reference for more extensive, larger-scale future studies. On the other hand, because complicated shapes of rugae can cause intra-observer differences in identification, the casts were evaluated by a single investigator in this study to improve the identification and the reliability.

## 5. Conclusions

The palatal rugae pattern has various traits that can be used to differentiate between ethnicities. According to the current study, the overall number of palatal rugae in the Sistani ethnicity is much lower than in the Turkmen and Fars ethnicities. In addition, the total number of palatal rugae in the Fars ethnicity was more on the left side of the palate than the right, and it was greater in women than men. Furthermore, in the Fars, the most prevalent palatal rugae shapes were wavy, straight, curved, and divergent; in the Turkmen, straight, wavy, and divergent; and in the Sistani, straight, wavy, and curved. As a result, the current study found significant disparities in the palatal rugae patterns among three Iranian ethnicities. Therefore, the palatal rugae can be employed as a complementary technique to human identification in forensic medicine. However, larger sample size investigations are needed to corroborate the findings of the current study.

## Figures and Tables

**Table 1:** Distribution of age and sex of the samples in ethnicities.

Variable	Ethnicity			*p* Value
	Fars	Turkmen	Sistani	
	N (%)	N (%)	N (%)	
**Sex**				0.500 *
Female	44 (42.72)	38 (36.89)	46 (44.66)	
Male	59 (57.28)	65 (63.11)	57 (55.34)	
**Age (mean ± SD)**	16.71 ± 3.25	16.56 ± 3.30	17.29 ± 2.97	0.230 **

* Calculated by chi-squared test; ** Calculated by one-way ANOVA test.

**Table 2:** Distribution of palatal rugae numbers on both sides of the palate in ethnicities by sex.

Side of Palate	Sex	Ethnicity			*p* Value *	*p* Value ** T vs. F	*p* Value ** T vs. S	*p* Value ** S vs. F
		Fars Mean ± SD	Turkmen Mean ± SD	Sistani Mean ± SD				
Right	Female	6.05 ± 1.33	6.2 ± 1.41	5.71 ± 1.38	0.152	-	-	-
	Male	5.52 ± 1.21	5.84 ± 1.26	5.65 ± 1.46	0.551	-	-	-
	Total	5.85 ± 1.30	6.08 ± 1.38	5.68 ± 1.41	0.113	-	-	-
Left	Female	6.50 ± 1.40	6.15 ± 1.24	5.70 ± 1.54	**0.008**	0.414	0.315	**0.008**
	Male	6.06 ± 1.31	6.18 ± 1.64	5.36 ± 1.48	**0.023**	0.990	**0.034**	0.139
	Total	6.26 ± 1.44	6.12 ± 1.43	5.52 ± 1.46	**<0.001**	0.990	**0.010**	**0.001**
Total	Female	12.55 ± 2.01	12.35 ± 2.16	11.42 ± 2.32	**0.011**	0.990	**0.017**	**0.017**
	Male	11.59 ± 2.03	12.02 ± 2.43	11.02 ± 2.30	0.126	-	-	-
	Total	12.11 ± 2.08	12.21 ± 2.27	11.16 ± 2.29	**0.001**	0.990	**0.002**	**0.007**

(F) Fars; (T) Turkmen; (S) Sistani. * Calculated by one-way analysis of variance (ANOVA) test; ** calculated by Tukey’s test; significant *p* values are shown in bold style.

**Table 3:** Distribution of different rugae shapes in ethnicities by sex.

Shape of Rugae	Sex	Ethnicity			*p* Value *	*p* Value ** T vs. F	*p* Value ** T vs. S	*p* Value ** S vs. F
		Fars Mean ± SD	Turkmen Mean ± SD	Sistani Mean ± SD				
**Straight**	Female	3.5 ± 1.54	4.16 ± 1.79	3.51 ± 1.87	0.056	-	-	-
	Male	3.06 ± 1.57	3.94 ± 1.73	3.17 ± 1.52	**0.033**	**0.040**	0.100	0.900
	Total	3.31 ± 1.56	4.08 ± 1.76	3.36 ± 1.72	**0.001**	**0.004**	**0.009**	0.900
**Wavy**	Female	3.72 ± 1.69	3.73 ± 1.54	3.28 ± 1.48	0.210	-	-	-
	Male	3.63 ± 1.43	3.59 ± 1.49	2.85 ± 1.40	**0.024**	0.900	**0.010**	**0.010**
	Total	3.68 ± 1.57	3.68 ± 1.52	3.10 ± 1.46	**0.008**	0.900	**0.003**	**0.008**
**Curved**	Female	2.29 ± 1.20	1.85 ± 0.95	2.46 ± 1.09	**0.020**	0.080	0.007	0.510
	Male	1.80 ± 1.01	1.71 ± 0.89	2.76 ± 1.44	**<0.001**	0.900	**0.001**	**0.002**
	Total	2.10 ± 1.15	1.80 ± 0.92	2.59 ± 1.25	**<0.001**	0.150	**<0.001**	**0.009**
**Circular**	Female	1.15 ± 0.37	1 ± 0	1.30 ± 0.48	0.231	-	-	-
	Male	1 ± 0	1.11 ± 0.33	1 ± 0	0.405	-	-	-
	Total	1.10 ± 0.31	1.06 ± 0.25	1.19 ± 0.40	0.486	-	-	-
**Angle**	Female	1.46 ± 0.70	1.25 ± 0.52	1.12 ± 0.35	0.280	-	-	-
	Male	1.12 ± 0.33	1.57 ± 0.81	1.12 ± 0.35	**0.024**	**0.010**	0.120	0.900
	Total	1.29 ± 0.57	1.39 ± 0.67	1.12 ± 0.34	0.280	-	-	-
**Cross**	Female	1.29 ± 0.46	1 ± 0	1 ± 0	0.063	-	-	-
	Male	1.2 ± 0.42	1.22 ± 0.44	1 ± 0	0.792	-	-	-
	Total	1.25 ± 0.44	1.1 ± 0.30	1 ± 0	0.163	-	-	-
**Diverging**	Female	1 ± 0	1.1 ± 0.31	1.10 ± 0.31	0.620	-	-	-
	Male	1.2 ± 0.44	1 ± 0	1 ± 0	0.320	-	-	-
	Total	1.07 ± 0.26	1.07 ± 0.26	1.07 ± 0.26	0.997	-	-	-
**Converging**	Female	1.13 ± 0.35	1 ± 0	1.07 ± 0.26	0.695	-	-	-
	Male	1 ± 0	1 ± 0	1.22 ± 0.44	0.289	-	-	-
	Total	1.08 ± 0.28	1 ± 0	1.13 ± 0.34	0.618	-	-	-
**Branching with divergence**	Female	1.69 ± 0.98	1.71 ± 0.88	1.64 ± 0.75	0.939	-	-	-
	Male	1.76 ± 0.80	1.80 ± 0.86	1.86 ± 0.91	0.892	-	-	-
	Total	1.73 ± 0.90	1.76 ± 0.87	1.71 ± 0.38	0.934	-	-	-
**Branching with convergence**	Female	1.31 ± 0.47	1.4 ± 0.64	1.15 ± 1.37	0.413	-	-	-
	Male	1.25 ± 0.68	1.27 ± 0.46	1.36 ± 0.49	0.807	-	-	-
	Total	1.28 ± 0.56	1.36 ± 0.59	1.28 ± 0.45	0.795	-	-	-
**Nonspecific**	Female	1 ± 0	6.02 ± 5.66	5.76 ± 5.96	0.389	-	-	-
	Male	1 ± 0	1 ± 0	9.56 ± 0.42	**<0.001**	-	-	-
	Total	1 ± 0	1 ± 0	1 ± 0	NR	-	-	-

(F) Fars; (T) Turkmen; (S) Sistani. * Calculated by one-way analysis of variance (ANOVA) test; ** calculated by Tukey’s test; significant *p* values are shown in bold style.

**Table 4:** Distribution of different rugae lengths in ethnicities by age.

Length	Age (Year)	Ethnicity			*p* Value *	*p* Value ** T vs. F	*p* Value ** T vs. S	*p* Value ** S vs. F
		Fars Mean ± SD	Turkmen Mean ± SD	Sistani Mean ± SD				
**Primary**	**<18**	9.44 ± 1.83	8.98 ± 1.59	8.67 ± 1.65	**0.020**	0.272	0.760	**0.017**
	**≥18**	9 ± 1.31	9.09 ± 1.64	8.79 ± 1.47	0.757	-	-	-
**Secondary**	**<18**	1.74 ± 1.18	2.17 ± 1.54	1.63 ± 1.35	**0.036**	0.153	**0.047**	0.990
	**≥18**	2.66 ± 1.52	2.19 ± 1.40	2 ± 1.73	0.304	-	-	-
**Fragmented**	**<18**	0.46 ± 0.65	0.67 ± 0.98	0.55 ± 0.87	0.319	-	-	-
	**≥18**	0.66 ± 0.70	0.61 ± 0.66	0.48 ± 0.82	0.647	-	-	-

(F) Fars; (T) Turkmen; (S) Sistani; (**<**) less than; (**≥**) more than or equal. * Calculated by one-way analysis of variance (ANOVA) test; ** calculated by Tukey’s test; significant *p* values are shown in bold style.

**Table 5:** Distribution of different rugae directions in ethnicities by sex.

Direction	Sex	Ethnicity			*p* Value *	*p* Value ** T vs. F	*p* Value ** T vs. S	*p* Value ** S vs. F
		Fars Mean ± SD	Turkmen Mean ± SD	Sistani Mean ± SD				
**Backward**	**Female**	6.37 ± 2.35	6.50 ± 2.58	6.26 ± 2.62	0.866	-	-	-
	**Male**	6.84 ± 2.72	7 ± 2.71	6.08 ± 2.69	0.247	-	-	-
	**Total**	6.57 ± 2.51	6.68 ± 2.62	6.18 ± 2.64	0.345	-	-	-
**Forward**	**Female**	3.32 ± 2.19	3.64 ± 2.36	2.49 ± 2.01	**0.014**	0.990	**0.013**	0.131
	**Male**	2.61 ± 1.93	3.34 ± 1.89	2.73 ± 1.70	0.171	-	-	-
	**Total**	3.01 ± 2.10	3.53 ± 2.19	2.60 ± 1.88	**0.005**	0.224	**0.004**	0.443
**Straight**	**Female**	3.08 ± 1.79	2.30 ± 1.51	2.75 ± 1.66	**0.034**	**0.030**	0.418	0.854
	**Male**	2.27 ± 1.93	2 ± 1.48	2.17 ± 1.58	0.763	-	-	-
	**Total**	2.73 ± 1.88	2.19 ± 1.50	2.49 ± 1.64	0.070	-	-	-

(F) Fars; (T) Turkmen; (S) Sistani. * Calculated by one-way analysis of variance (ANOVA) test; ** calculated by Tukey’s test; significant *p* values are shown in bold style.

## Data Availability

The data supporting the results in this study are available upon request from the first corresponding author (M.P.).

## References

[1] Higgins D., Austin J.J. (2013). Teeth as a source of DNA for forensic identification of human remains: A Review. Sci. Justice.

[2] Dumache R., Ciocan V., Muresan C., Enache A. (2016). Molecular DNA Analysis in Forensic Identification. Clin. Lab..

[3] Oldoni F., Podini D. (2019). Forensic molecular biomarkers for mixture analysis. Forensic Sci. Int. Genet..

[4] Oldoni F., Kidd K.K., Podini D. (2019). Microhaplotypes in forensic genetics. Forensic Sci. Int. Genet..

[5] Krishan K., Kanchan T., Garg A.K. (2015). Dental Evidence in Forensic Identification—An Overview, Methodology and Present Status. Open Dent. J..

[6] Malekzadeh A.R., Pakshir H.R., Ajami S., Pakshir F. (2018). The Application of Palatal Rugae for Sex Discrimination in Forensic Medicine in a Selected Iranian Population. Iran. J. Med. Sci..

[7] Smitha T., Sheethal H.S., Hema K.N., Franklin R. (2019). Forensic odontology as a humanitarian tool. J. Oral Maxillofac. Pathol..

[8] de La Dure-Molla M., Fournier B.P., Manzanares M.C., Acevedo A.C., Hennekam R.C., Friedlander L., Boy-Lefèvre M.L., Kerner S., Toupenay S., Garrec P. (2019). Elements of morphology: Standard terminology for the teeth and classifying genetic dental disorders. Am. J. Med. Genet. A.

[9] Madi H.A., Swaid S., Al-Amad S. (2013). Assessment of the uniqueness of human dentition. J. Forensic Odonto-Stomatol..

[10] Tinoco R.L., Martins E.C., Daruge E., Daruge E., Prado F.B., Caria P.H. (2010). Dental anomalies and their value in human identification: A case report. J. Forensic Odonto-Stomatol..

[11] Kiran R., Chapman J., Tennant M., Forrest A., Walsh L.J. (2019). Detection of Tooth-Colored Restorative Materials for Forensic Purposes Based on Their Optical Properties: An In Vitro Comparative Study. J. Forensic Sci..

[12] Pramod J.B., Marya A., Sharma V. (2012). Role of forensic odontologist in post mortem person identification. Dent. Res. J..

[13] Mishra S.K., Mahajan H., Sakorikar R., Jain A. (2014). Role of prosthodontist in forensic odontology. A literature review. J. Forensic Dent. Sci..

[14] Bathala L.R., Rachuri N.K., Rayapati S.R., Kondaka S. (2016). Prosthodontics an “arsenal” in forensic dentistry. J. Forensic Dent. Sci..

[15] Chugh A., Narwal A. (2017). Oral mark in the application of an individual identification: From ashes to truth. J. Forensic Dent. Sci..

[16] Muthusubramanian M., Limson K.S., Julian R. (2005). Analysis of rugae in burn victims and cadavers to simulate rugae identification in cases of incineration and decomposition. J. Forensic Odonto-Stomatol..

[17] Caldas I.M., Magalhães T., Afonso A. (2007). Establishing identity using cheiloscopy and palatoscopy. Forensic Sci. Int..

[18] Nuzzolese E., Lusito S., Solarino B., Di Vella G. (2008). Radiographic dental implants recognition for geographic evaluation in human identification. J. Forensic Odonto-Stomatol..

[19] Ziętkiewicz E., Witt M., Daca P., Zebracka-Gala J., Goniewicz M., Jarząb B., Witt M. (2012). Current genetic methodologies in the identification of disaster victims and in forensic analysis. J. Appl. Genet..

[20] Dutta S.R., Singh P., Passi D., Varghese D., Sharma S. (2016). The Role of Dentistry in Disaster Management and Victim Identification: An Overview of Challenges in Indo-Nepal Scenario. J. Maxillofac. Oral Surg..

[21] Morgan O.W., Sribanditmongkol P., Perera C., Sulasmi Y., Van Alphen D., Sondorp E. (2006). Mass fatality management following the South Asian tsunami disaster: Case studies in Thailand, Indonesia, and Sri Lanka. PLoS Med..

[22] Suwalowska H., Amara F., Roberts N., Kingori P. (2021). Ethical and sociocultural challenges in managing dead bodies during epidemics and natural disasters. BMJ Glob. Health.

[23] de Boer H.H., Roberts J., Delabarde T., Mundorff A.Z., Blau S. (2020). Disaster victim identification operations with fragmented, burnt, or commingled remains: Experience-based recommendations. Forensic Sci. Res..

[24] Forrest A. (2019). Forensic odontology in DVI: Current practice and recent advances. Forensic Sci. Res..

[25] Yoon S., Jain A.K. (2015). Longitudinal study of fingerprint recognition. Proc. Natl. Acad. Sci. USA.

[26] Prinz M., Carracedo A., Mayr W.R., Morling N., Parsons T.J., Sajantila A., Scheithauer R., Schmitter H., Schneider P.M. (2007). DNA Commission of the International Society for Forensic Genetics (ISFG): Recommendations regarding the role of forensic genetics for disaster victim identification (DVI). Forensic Sci. Int. Genet..

[27] de Boer H.H., Blau S., Delabarde T., Hackman L. (2019). The role of forensic anthropology in disaster victim identification (DVI): Recent developments and future prospects. Forensic Sci. Res..

[28] Holder E.H., Robinson L.O., Laub J.H. (2011). The Fingerprint Sourcebook.

[29] Hinchliffe J. (2011). Forensic odontology, part 2. Major disasters. Br. Dent. J..

[30] Setiadi D.S., Syukriani Y.F., Supian S., Oscandar F., Malinda Y., Nugraha A. (2019). Association between Direction Patterns of Palatal Rugae and Thumbprints: Implications for Forensic Identification. J. Dent. Indones..

[31] Suhartono A.W., Syafitri K., Puspita A.D., Soedarsono N., Gultom F.P., Widodo P.T., Luthfi M., Auerkari E.I. (2016). Palatal rugae patterning in a modern Indonesian population. Int. J. Leg. Med..

[32] Pakshir F., Ajami S., Pakshir H.R., Malekzadeh A.R. (2019). Characteristics of Palatal Rugae Patterns as a Potential Tool for Sex Discrimination in a Sample of Iranian Children. J. Dent..

[33] Hosmani J., Gadekar N.B., Kotrashetti V.S., Nayak R., Babji D., Mishra S. (2018). Comparison of palatal rugae pattern among Indian and Tibetan population. J. Forensic Dent. Sci..

[34] Ramdas S., Bommanavar S., Baad R., Vibhute N., Belgaumi U., Kadashetti V., Kamate W. (2019). Correlation and comparison of dactyloscopy and palatoscopy with blood groups among dental students from Western Maharashtra. Med. J. Dr. D. Y. Patil Vidyapeeth.

[35] Buyuk S.K., Simsek H., Yasa Y., Genc E., Turken R. (2019). Morphological assessment of palatal rugae pattern in a Turkish subpopulation. Aust. J. Forensic Sci..

[36] Thomas C.J., Kotze T.J. (1983). The palatal rugae pattern: A new classification. J. Dent. Assoc. S. Afr..

[37] Ahmed A.A., Hamid A. (2015). Morphological study of palatal rugae in a Sudanese population. Int. J. Dent..

[38] Sheikhi M., Zandi M., Ghazizadeh M. (2018). Assessment of palatal rugae pattern for sex and ethnicity identification in an iranian population. Dent. Res. J..

[39] Saadeh M., Ghafari J.G., Haddad R.V., Ayoub F. (2017). Palatal rugae morphology in an adult mediterranean population. J. Forensic Odonto-Stomatol..

[40] Dos Santos K.C., Fernandes C.M.S., da Costa Serra M. (2011). Evaluation of a digital methodology for human identification using palatal rugoscopy. Braz. J. Oral Sci..

[41] Kommalapati R.K., Katuri D., Kattappagari K.K., Kantheti L.P.C., Murakonda R.B., Poosarla C.S., Chitturi R.T., Gontu S.R., Baddam V.R.R. (2017). Systematic Analysis of Palatal Rugae Pattern for Use in Human Identification between Two Different Populations. Iran. J. Public Health.

[42] Gupta A.A., Kheur S., Alshehri A., Awadh W., Ahmed Z.H., Feroz S.M., Khan S.S., Mushtaq S., Dewan H., Khurshid Z. (2022). Is Palatal Rugae Pattern a Reliable Tool for Personal Identification following Orthodontic Treatment? A Systematic Review and Meta-Analysis. Diagnostics.

[43] Kolude B., Akinyele A., Joshua O.T., Ahmed L. (2016). Ethnic and gender comparison of rugae patterns among clinical dental trainees in Ibadan, Nigeria. Pan Afr. Med. J..

[44] Shailaja A.M., Romana I.R.U., Narayanappa G., Smitha T., Gowda N.C., Vedavathi H.K. (2018). Assessment of palatal rugae pattern and its significance in orthodontics and forensic odontology. J. Oral Maxillofac. Pathol..

[45] Savita J.K., Yathindra Kumar B.N., Satish G., Divya K.T., Ranjitha J., Pujari R.K. (2016). Prevalence of palatal rugae shapes in Karnataka and Kerala population: A cross-sectional study. J. Int. Soc. Prev. Community Dent..

[46] Sherif A.F., Hashim A.A., Al Hanafy M.A., Soliman E.M. (2018). A pilot- cross sectional study of palatal Rugae shape and direction among Egyptians and Malaysians. Egypt. J. Forensic Sci..

[47] Saberi R. (2019). Ethnic enclosure in multicultural Muslim community life: Case study in Golestan Province, IR Iran. J. Studi Sos. Dan Polit..

[48] Majbouri M., Fesharaki S. (2019). Iran’s Multi-ethnic Mosaic: A 23-Year Perspective. Soc. Indic. Res..

[49] Semnani S., Sadjadi A., Fahimi S., Nouraie M., Naeimi M., Kabir J., Fakheri H., Saadatnia H., Ghavamnasiri M.R., Malekzadeh R. (2006). Declining incidence of esophageal cancer in the Turkmen Plain, eastern part of the Caspian Littoral of Iran: A retrospective cancer surveillance. Cancer Detect. Prev..

[50] Hassan H.D. (2008). Iran: Ethnic and Religious Minorities.

[51] Hemanth M., Vidya M., Shetty N., Karkera B.V. (2010). Identification of individuals using palatal rugae: Computerized method. J. Forensic Dent. Sci..

[52] Shetty D., Juneja A., Jain A., Khanna K.S., Pruthi N., Gupta A., Chowdhary M. (2013). Assessment of palatal rugae pattern and their reproducibility for application in forensic analysis. J. Forensic Dent. Sci..

[53] Abdel-Aziz H.M., Sabet N.E. (2001). Palatal rugae area: A landmark for analysis of pre- and post-orthodontically treated adult Egyptian patients. East. Mediterr. Health J..

[54] Kar S., Tripathi A., Madhok R. (2016). Replication of Palatal Rugae and Incorporation in Complete Denture. J. Clin. Diagn. Res..

[55] Patil M.S., Patil S.B., Acharya A.B. (2008). Palatine Rugae and Their Significance in Clinical Dentistry: A Review of the Literature. J. Am. Dent. Assoc..

[56] Shanmugam S., Anuthama K., Shaikh H., Murali K., Suresan V., Nisharudeen K., Devi S.P.B., Rajasundaram P. (2012). Palatal rugae in population differentiation between South and North Indians: A discriminant function analysis. J. Forensic Dent. Sci..

[57] De Angelis D., Riboli F., Gibelli D., Cappella A., Cattaneo C. (2012). Palatal rugae as an individualising marker: Reliability for forensic odontology and personal identification. Sci. Justice.

[58] Arora V., Bagewadi A., Keluskar V., Shetti A. (2008). Comparison of palatal rugae pattern in two populations of India. Int. J. Med. Toxicol. Leg. Med..

[59] Kashima K. (1990). Comparative study of the palatal rugae and shape of the hard palatal in Japanese and Indian children. Aichi Gakuin Daigaku Shigakkai Shi.

[60] Kapali S., Townsend G., Richards L., Parish T. (1997). Palatal rugae patterns in Australian Aborigines and Caucasians. Aust. Dent. J..

[61] Selvamani M., Hosallimath S., Madhushankari, Basandi P.S., Yamunadevi A. (2015). Dimensional and morphological analysis of various rugae patterns in Kerala (South India) sample population: A cross-sectional study. J. Nat. Sci. Biol. Med..

[62] Kalyani K.R., Kumar K.K., Sekhar P.C., Reddy G.S., Prasad L.K., Reddy B.V.R. (2016). Analysis of palatal rugae patterns among two ethnic populations of Andhra Pradesh. J. NTR Univ. Health Sci..

[63] Ibeachu P., Didia B., Arigbede A. (2014). A comparative study of palatal rugae patterns among igbo and ikwerre ethnic groups of Nigeria: A University of Port Harcourt Study. Anat. Res. Int..

[64] Chong J.A., Mohamed A.M.F.S., Pau A. (2020). Morphological patterns of the palatal rugae: A review. J. Oral Biosci..

[65] Surekha R., Anila K., Reddy V.S., Hunasgi S., Ravikumar S., Ramesh N. (2012). Assessment of palatal rugae patterns in Manipuri and Kerala population. J. Forensic Dent. Sci..

[66] Bajracharya D., Vaidya A., Thapa S., Shrestha S. (2013). Palatal rugae pattern in nepalese subjects. Orthod. J. Nepal.

[67] Mohamed T.J. (2016). A comparison of rugae pattern in males and females as a samples of Iraqi population. Tikrit J. Dent. Sci..

[68] Saini A., Garg A. (2018). A demographic study of palatal rugae patterns among North and North East Indian populations. Int. J. Forensic Odontol..

[69] Silva-Sousa A.C., Marañón-Vásquez G.A., Gerber J.T., Judachesci C.S., Stuani M.B.S., Matsumoto M.A.N., Coletta R.D., Scariot R., Küchler E.C. (2020). Left-right asymmetry in palatal rugae is associated with genetic variants in WNT signaling pathway. Arch. Oral Biol..

[70] Alokaili A.G., Sedeeq B.I., Hadi R., Saleh Z. (2018). Comparative Study of Palatal Rugae Patterns in Two Populations (Arabs-Asian and Indians). Tikrit J. Dent. Sci..

[71] Nayak P., Acharya A.B., Padmini A., Kaveri H. (2007). Differences in the palatal rugae shape in two populations of India. Arch. Oral Biol..

[72] Chandra S., Pandey V., Wasti A., Mangat S.S., Bhagat J.K., Singh R. (2016). Analysis of rugae pattern in Ranchi and Patna population. J. Int. Oral Health.

[73] Azab S.M.S., Magdy R., El Deen M.A.S. (2016). Patterns of palatal rugae in the adult Egyptian population. Egypt. J. Forensic Sci..

[74] Kalia K.P. (2005). Palatal rugae pattern in Mysorean and Tibetan populations. J. Dent. Res..

[75] Dohke M., Osato S. (1994). Morphological study of the palatal rugae in Japanese. Jpn. J. Oral Biol..

[76] Mattoo K.A., Darraj A., Rasean D.A., Majali R., Shagri S. (2019). Analysis of rugae pattern morphology and their comparative differences in a Jazan population sample. Saudi. J. Forensic Med. Sci..

